# Chromosome-scale genome assembly of *Eustoma grandiflorum*, the first complete genome sequence in the genus *Eustoma*

**DOI:** 10.1093/g3journal/jkac329

**Published:** 2022-12-19

**Authors:** Kenta Shirasawa, Ryohei Arimoto, Hideki Hirakawa, Motoyuki Ishimori, Andrea Ghelfi, Masami Miyasaka, Makoto Endo, Saneyuki Kawabata, Sachiko N Isobe

**Affiliations:** Kazusa DNA Research Institute, Kazusa-Kamatari, 2-6-7, Kisarazu, Chiba 292-0818, Japan; Takii & Co., Ltd., Hari 1360, Konan, Shiga 520-3231, Japan; Kazusa DNA Research Institute, Kazusa-Kamatari, 2-6-7, Kisarazu, Chiba 292-0818, Japan; Graduate School of Agricultural and Life Sciences, The University of Tokyo, 1-1-1, Yayoi, Bunkyo-Ku, Tokyo 113-8657, Japan; Kazusa DNA Research Institute, Kazusa-Kamatari, 2-6-7, Kisarazu, Chiba 292-0818, Japan; Bioinformation and DDBJ Center, National Institute of Genetics, 1111 Yata, Mishima, Shizuoka 411-8540, Japan; Nagano Vegetable and Ornamental Crops Experiment Station, 1066-1 Soga, Shiojiri City, Nagano 399-6461, Japan; Takii & Co., Ltd., Hari 1360, Konan, Shiga 520-3231, Japan; Institute for Sustainable Agro-ecosystem Services, Graduate School of Agricultural and Life Sciences, The University of Tokyo, 1-1-1, Midori, Nishitokyo-Shi, Tokyo 18-0002, Japan; Kazusa DNA Research Institute, Kazusa-Kamatari, 2-6-7, Kisarazu, Chiba 292-0818, Japan

**Keywords:** *Eustoma grandiflorum*, genome assembly, gene prediction, genetic diversity

## Abstract

*Eustoma grandiflorum* (Raf.) Shinn. is an annual herbaceous plant native to the southern United States, Mexico, and the Greater Antilles. It has a large flower with a variety of colors and is an important flower crop. In this study, we established a chromosome-scale de novo assembly of *E. grandiflorum* genome sequences by integrating four genomic and genetic approaches: (1) Pacific Biosciences (PacBio) Sequel deep sequencing, (2) error correction of the assembly by Illumina short reads, (3) scaffolding by chromatin conformation capture sequencing (Hi-C), and (4) genetic linkage maps derived from an F_2_ mapping population. Thirty-six pseudomolecules and 64 unplaced scaffolds were created, with a total length of 1,324.8 Mb. A total of 36,619 genes were predicted on the genome as high-confidence genes. A comparison of genome structure between *E. grandiflorum* and *C. canephora* or *O. pumila* suggested whole-genome duplication after the divergence between the families Gentianaceae and Rubiaceae. Phylogenetic analysis with single-copy genes suggested that the divergence time between Gentianaceae and Rubiaceae was 74.94 MYA. Genetic diversity analysis was performed for nine commercial *E. grandiflorum* varieties bred in Japan, from which 254,205 variants were identified. This first report on the construction of a reference genome sequence in the genus *Eustoma* is expected to contribute to genetic and genomic studies in this genus and in the family Gentianaceae.

## Introduction

*Eustoma grandiflorum* (Raf.) Shinn., commonly known as Lisianthus, prairie gentian, or bluebell gentian, is an annual herbaceous plant native to the southern United States, Mexico, and the Greater Antilles (Shinners, 1957; Cruz-Duque *et al.* 2017). It has large flowers with a variety of colors, including white, pink, yellow, purple, and purple-edged white (Ohkawa and Sasaki, 1997). *E. grandiflorum* is cultivated around the world and has become one of the 10 most popular cut flowers (Azadi *et al.* 2016). It is an important flower crop especially in Japan, ranking fourth in production value in 2017 and third in cultivation area in 2018 (Onozaki *et al.* 2020). Numerous varieties have been bred in the commercial and public sectors (Ohkawa and Sasaki, 1997) as both selfed and F_1_ hybrids.

Genus *Eustoma*, which belongs to the family Gentianaceae and the tribe Chironiae, is small, comprising only three species: *E. grandiflorum*, *E. barkleyi* Standely, and *E. exaltatum* (L.) Salisb. Ex Don. (Barba-Gonzalez *et al.* 2015). *E. grandiflorum* was previously called *E. russellianum* and is sometimes classified as a subspecies of *E. exaltatum* (Turner 2014). In the NCBI taxonomy database (https://www.ncbi.nlm.nih.gov/taxonomy), *E. grandiflorum* is registered as a heterotypic synonym of *E. exaltatum* subsp. *russellianum* (Taxonomy ID: 52518). *E. grandiflorum* was previously considered an octoploid (Rork 1949), but a recent study suggested that *E. grandiflorum* is a diploid, with a chromosome number of 2n = 2X = 72 (Kawakatsu *et al.* 2021).

The family Gentianaceae consists of 6 tribes, 99 genera, and approximately 1,736 species (Struwe 2014). The family name Gentianaceae is derived from Gentius (181–168 Bc), an Illyrian king who reigned in ancient Greece, and who discovered the anti-inflammatory and other medical properties of gentian. As indicated by the origin of the family name, several species in the family, such as *Gentiana trifloral* (gentian) and *Swertia japonica*, have been used as medicinal or herbal plants. Chloroplast and plastid genome sequences were reported for several species in the genus *Gentiana* (Fu *et al.* 2016; Ni *et al.* 2016; Huang *et al.* 2019; Sun *et al.* 2019) and *Pterygocalyx* (Wang *et al*. 2019; Zou *et al*. 2021). Recently, the chromosome-level genome assembly of *G. dahurica* was reported as the first reference genome in the family Gentianaceae (Li *et al.* 2022).

In this study, we established a chromosome-scale de novo assembly of *E. grandiflorum* genome sequences. The assembled genome and predicted genes were compared with those of other species, including those in the order Gentianales, to assess the genome structure divergence and phylogenetic relations. This is the first report on the construction of reference genome sequences in *E*. *grandiflorum* as well as in the genus *Eustoma*. We expect that the assembled genome will contribute to advances in the research and breeding of *E. grandiflorum.* In addition, this is only the second report of a chromosome-level genome sequence assembly in this family. Finally, our results should lead to insights into the genome structure of the genus Eustoma and the family Gentianaceae.

## Materials and methods

### Whole-genome sequencing and assembly

An *E. grandiflora* inbred line, 10B-620, bred at the Nagano Vegetable and Ornamental Crops Experimental Station, was used for whole-genome sequencing with Illumina short reads and PacBio long reads. The genomic DNA was extracted from young leaves with the use of a Genomic DNA Extraction Column (Favorgen Biotech Corp., Ping-Tung, Taiwan) for short reads and a Genomic-tips Kit (Qiagen, Germantown, MD, USA) for long reads. An Illumina paired-end (PE) library was constructed with an expected insert size of 500 bp. Library sequencing was performed by an Illumina HiSeq system (Illumina, San Diego, CA, USA) with a read length of 101 nt (Supplementary Table 1). The genome size for 10B-620 was estimated based on kmer-frequency analysis with short reads by using Jellyfish ver. 2.1.1 (Marçais and Kingsford 2011). A long-read sequence library was prepared using the SMRTbell Express Template Prep Kit 1.0 (PacBio, Menlo Park, CA, USA). The size selection of the library was performed by BluePippin (Sage Science, Beverly, MA, USA) to remove DNA fragments less than 15 kb in length, and the library was then sequenced using the Sequel system (PacBio) with 14 SMRT cells.

The sequence reads were assembled using FALCON Unzip v.1.8.1 (Chin *et al.* 2016) with default parameters, and the generated primary contig sequences were polished twice using ARROW ver. 2.2.1 implemented in SMRT Link v.5.0 (PacBio). Illumina PE reads were then used for further error correction of the contig sequences with Pilon 1.22 (Walker *et al.* 2014).

### Linkage map construction

An F_2_ mapping population named 10B-58 was developed from reciprocal crosses between 10B-620 and an inbred *E. grandiflorum* line, 10B-503. The number of F_2_ individuals used to construct the linkage map was 104. Variants (SNPs and indels) segregating in the F_2_ population were detected by sequencing the dd-RAD-Seq and GRAS-Di libraries. Library construction was performed according to Shirasawa *et al.* for the dd-RAD-Seq (Shirasawa *et al.* 2016) and according to Miki *et al.* for GRAS-Di (Miki *et al.* 2020). Both libraries were sequenced using Illumina Hiseq 2000 (Illumina). A variant call was performed by bcftools 0.1.19 in Samtools with the following option: mpileup -d 10000000 -D -u -f (Li *et al*. 2009).

Segregate linkage maps were constructed by using Lep-MAP3 (Rastas 2017) and MSTmap (Wu *et al.* 2008). The map created using Lep-MAP3 was constructed with the variants identified on the FALCON-unzip contigs and was used to split misassembled contig sequences by comparing the SNP positions on the contigs with those on the linkage groups (LGs). The default parameters were used in the Lep-Map3, and the male map positions are shown in this study. The map created using MSTmap was constructed for revision of the chromosome-scale scaffolds after the Hi-C analysis. The following parameters were used to construct the linkage map: distance_function = kosambi, cut_off_*P*_value = 1e-12, no_map_distance = 20, no_map_size = 2, missing_threshold = 0.2, estimation_before_clustering = yes, detect_bad_data = no, objective_function = COUNT.

### Hi-C scaffolding and construction of chromosome-level scaffolds

A Hi-C library was constructed from young leaves of 10B-620 using the Proximo Hi-C Plant Kit (Phase Genomics, Seattle, WA, USA). The library was sequenced by Illumina NextSeq500, and the obtained PE reads were aligned onto the scaffolds by BWA (Li and Durbin 2009). Chromosome-scale scaffolds were created by using the Proximo Hi-C genome scaffolding platform (Phase Genomics) using a method similar to that described by Bickhart *et al.* (Bickhart *et al.* 2017). Juicebox (Durand *et al.* 2016) was then used to correct scaffolding errors. After error correction, the Hi-C scaffolds were cut and reordered by using ALLMAPS (Tang *et al.* 2015) and Ragoo (Alonge *et al*. 2019) with the MST map as a reference, and the chromosome-level scaffold sequences were determined.

Assembly quality was assessed by benchmarking universal single-copy ortholog (BUSCO) sequences using BUSCO v3.0 (Simão *et al.* 2015). Repetitive sequences in the assembled genome were identified by RepeatMasker 4.0.7 (http://www.repeatmasker.org/RMDownload.html) for known repetitive sequences registered in Repbase (https://www.girinst.org/repbase/) and de novo repetitive sequences defined by RepeatModeler 1.0.11 (http://www.repeatmasker.org/RepeatModeler).

### Transcriptome sequencing and gene prediction

Total RNAs were extracted from young leaves and buds of 10B-620 by using the RNeasy Plant Mini Kit RNA (Qiagen). Iso-Seq libraries were created for leaves and buds in accordance with the manufacturer's protocol (PacBio) and sequenced by a Sequel system with two SMRT cells. The obtained reads were clustered using the Iso-Seq 2 pipeline implemented in SMRT Link ver.5.1.0 (PacBio). The high-quality (hq) Iso-seq sequences were then mapped onto the assembled genome with Minimap2 (Li 2018) and collapsed to obtain nonredundant isoform sequences using a module in Cupcake ToFU (https://github.com/Magdoll/cDNA_Cupcake). Open reading frame (ORF) prediction on the collapsed sequences was performed using ANGEL (https://github.com/PacificBiosciences/ANGEL). Redundant sequences were then removed by the CD-HIT program (Fu *et al.* 2012), and nonredundant complete confidence (cc) sequences were mapped onto the assembled genome sequences by GMAP ver. 2020.06.01 (Wu and Watanabe 2005).

Empirical gene prediction was performed for the repeat masked assembled genome sequences by BRAKER2 (Brůna *et al*. 2021) with published *E. grandifolum* transcript sequences (Supplementary Table 2). After the removal of the redundant variant sequences, the gene sequences predicted by BRAKER2 were merged with those mapped with cc Iso-Seq sequences. When gene sequences were predicted by both BRAKER v2 and Iso-Seq, the longest coding sequences (CDSs) were selected.

To classify the predicted gene sequences based on the evidence level, a similarity search was performed against the NCBI NR protein database (http://www.ncbi.nlm.nih.gov) and UniProtKB (https://www.uniprot.org) using DIAMOND (Buchfink *et al.* 2015) with 60% ≤ similarity, 50% ≤ mapped length ≤ 150%, and E-value ≤ 1E-80. BLASTP searches were also performed for the gene sequences of *Vitis vinifera* (12X) (Jaillon *et al.* 2007) and *Arabidopsis thaliana* (Araport 11) (Cheng *et al.* 2017) with 30% ≤ similarity (*V. vinifera*) or 25% ≤ similarity (*A. thaliana*), 50% ≤ mapped length ≤ 150%, and E-value ≤ 1E-80. Domains were searched by HAMMER v3.3.2 (http://hmmer.org/) with E-value ≤ 1E-30, and TPM values were calculated by Salmon (Patro *et al.* 2017) with the RNA-Seq reads listed in Supplementary Table 2. The high-confidence (HC) gene sequences were selected under the following conditions: TPM value > 0.2, identified protein domain sequences, gene sequence hits in the UniProtKB or NR protein database, or *V. vinifera* genes. Transposon elements (TEs) were classified based on the results of similarity searches against UniProtKB. The gene sequences not classified as HC or TEs were classified as LC (low confidence). Functional gene annotation was also performed by using a modified version of the Hayai annotation (Ghelfi *et al*. 2019), called ZenAnnotation (https://github.com/aghelfi/ZenAnnotation), into which OrthoDB (https://www.orthodb.org/) sequences were incorporated in order to allow contaminant detection. The parameters for sequence alignment, performed by DIAMOND with a more-sensitive mode, were sequence identity 50%, query cover 50%, and subject cover 50%.

### Comparison with other plant species at the genome and gene levels

The genome sequence comparison was performed by using D-Genies (Cabanettes and Klopp 2018). The predicted gene sequences were clustered by the OrthoFinder v2.5 (Emms and Kelly 2019) with those of *G. dahurica* (Gda, Li *et al.* 2022), *O. pumila* (Opu_r1.4, Rai *et al.* 2021), *C. canephora* (v1, Denoeud *et al.* 2014), *V. vinifera* (12X). and *A. thaliana* (Araport 11). The sequence alignments were performed for each cluster including single-copy genes among the six species by MUSCLE v3.8.1551 (Edgar 2004). The conserved sequences were detected by Gblocks v0.91b (Talavera *et al*. 2007) and were aligned by ClustalW2 (Larkin *et al*. 2007).

The divergence time was inferred by using MEGA7 (Kumar *et al*. 1994) according to the results of the ClustalW2 alignment. A divergence time of 115 MYA between *C. canephora* and *V. vinifera* registered in TIMETREE (Kumar *et al.* 2017) was used for calibration. Ks values were calculated using a KaKs calculator (Wang *et al.* 2010).

### Diversity analysis in nine commercial varieties

Genetic diversity was investigated in nine commercial varieties bred in Japan: Yukitemari, Borelo white, Paleo pink, Exe lavender, La folia, Umi honoka, Robera clear pink, Korezo light pink, and Cereb pink. Plant materials were grown in a field at Takii Co. (Shiga, Japan), and the genomic DNA of each was extracted from young leaves with the use of a Genomic DNA Extraction Column (Favorgen Biotech Corp). Whole-genome shotgun (WGS) sequencing was performed by using Illumina Hisex X (Illumina) with 150 PE reads. The WGS reads were mapped onto the assembled genome sequences by using Bowtie 2 (Langmead and Salzberg 2012), and base variants were identified using bcftools 0.1.19 mpileup in Samtools (Li *et al.* 2009). Genetic distances were calculated by using the Distance Matrix function in TASSEL 5 (Bradbury *et al*. 2007). A neighbor-joining phylogenetic tree was constructed using MEGA ver 10.1.8 (Kumar *et al*. 1994).

## Results and discussion

### Estimation of 10B-620 genome size

WGS reads were obtained for an *E. grandiflora* inbred line, 10B-620, with a total length of 64.26 Gb reads (Supplementary Table 1). The distribution of distinct *k*-mers (k = 17) shows a single large peak at multiplicities of 34, suggesting that 10B-620 was a highly homozygous material (Supplementary Figure 1). Based on the identified peak, the genome size of 10B-620 was estimated to be 1587.6 Mb. Lindsay *et al*. (1994) reported that the nuclear DNA content of diploid *E. grandiflorum* was 3.26 ± 0.10 pg DNA per 2C, suggesting that the total base length of the genome was 1512.64 Mb per C. Our genome size estimation was similar to that in the previous report.

### PacBio assembly

A total length of 104.47 Gb of PacBio reads was generated from 14 SMRT cells. The obtained read coverage against the 10B-620 genome was 65.8x. The Falcon-unzip assembly generated 675 primary and 7,724 haplotig contigs (Supplementary Table 3). The total length of the primary contigs was 1322.4 Mb, occupying 83.2% of the estimated genome size (1,587.6 Mb). Meanwhile, 7,724 haplotig contigs were created with a total length of 326.9 Mb. The sum of the primary and haplotig sequences was 1,649.3 Mb, which exceeded the estimated genome size. Because 10B-620 was considered a highly homozygous material, there was a possibility that the haplotig contigs were derived from repetitive sequence regions in a haploid genome, not from heterozygous sequences between the haploids. However, because it was difficult to distinguish between heterozygous and repetitive sequences in the haplotig contigs, we proceeded with the analysis using only the primary sequence. The primary contig sequences were polished with Sequel reads using Arrow, followed by further polishing with the Illumina reads using Pilon. The resultant number of primary contig sequences was 616, with a total length of 1324.7 Mb.

### Linkage map construction for identification of misassembly of the PacBio contigs

A genetic linkage map was roughly constructed to investigate misassembly of the primary contigs. dd-RAD-Seq and GRAS-Di sequences were obtained for the 104 F_2_ population (10B-58) derived from crosses between 10B-620 and 10B-503. The reads were mapped onto the 616 primary sequences, resulting in the identification of 20,401 (dd-RAD-Seq) and 5,488 (GRAS-Di) base variants. The variants identified from the two libraries were then merged, and a linkage map was constructed by Lep-MAP3. A total of 20 LGs were generated, with a total length of 2,331.5 cM (Supplementary Figure 2). The numbers of mapped loci and bins (unique positions of loci) were 17,872 and 1,358, respectively.

The number of LGs of the linkage map created by Lep-Map3 (hereinafter the Lep-map), was less than expected, i.e. 36, suggesting the possibility that variants derived from multiple chromosomes were mapped onto a single LG. However, it was considered that the Lep-map reflected the correct alignment of the variants on the chromosomes at the local level. Therefore, we used the Lep-map to detect possible misassembly on the primary contigs in regard to F2 genotype segregation patterns to avoid reflecting errors on the Lep-map side. A total of 79 contigs were identified as possible misassemblies by comparing the variant positions between the Lep-map and F_2_ genotype segregation patterns. The 79 contigs were split at the points of possible misassembly, and the resultant 753 primary contigs were used for subsequent Hi-C scaffolding (Supplementary Table 3).

### Chromosome-level scaffolding with Hi-C reads and a linkage map

Several different chromosome numbers and ploidies of *E. grandiflorum* have been reported. For example, Rork (1949) described *E. grandiflorum* as an octoploid, with a chromosome number of 2n = 8X = 72 based on observation of chromosomes in root chips. Griesbach and Bhat (1990) reported that *E. grandiflorum* had 18 basic chromosomes according to a chromosome observation in meiotic metaphase I of diploid *E. grandiflorum*. Later, Kawakatsu *et al.* (2021) suggested that *E. grandiflorum* had 2n = 2x = 72 chromosomes based on the result of SSR linkage map construction. *E. exaltatum* was also considered to have 2n = 2x = 72 chromosomes (Barba-Gonzalez *et al.* 2015). Hence, we constructed chromosome-level scaffolds under the assumption that *E. grandiflorum* had 36 basic chromosomes.

A total of 589.9 M Hi-C reads were generated and used for scaffolding of the 753 primary contigs with N100. One hundred scaffolds were generated, including 36 chromosome-level scaffolds (Supplementart Figure S3A). The total length of the 100 scaffolds was 1,324.8 Mb (Supplementary Table 3), and the 36 chromosome-level scaffolds occupied 98.8% of the total length.

To identify possible misassemblies on the Hi-C scaffolds, a linkage map was reconstructed by using MST map software (here, we call the resulting linkage map an MST map). The dd-RAD-Seq and GRAS-Di sequences of the F_2_ population were mapped onto the 100 HI-C scaffold sequences. The variants were filtered-out with DP ≥ 10 and GQ ≥ 50, and 6,430 variants on the 36 chromosome-level scaffolds were mapped onto 43 LGs. The median reads coverage of the filtered-out variants was 26.7. The number of created LGs was 43, which was larger than the expected number of 36 reads, suggesting that individual chromosomes may have split into multiple LGs. Meanwhile, the correspondence between LG and scaffolds and the F2 genotype segregation pattern suggested that there were cases in which sequences derived from multiple chromosomes were assembled into a single scaffold (Supplementary Table 4 and Supplementary Table 5). Therefore, we realigned the primary contigs that had been scaffolded on the wrong position by Hi-C analysis by referring to the base variant positions on the MST map.

The positions corresponding to the 6,430 variants on the MST map were determined on 375 of the 753 primary contigs. The 375 primary contigs were then aligned on the 43 LGs of the MST map by using ALLMAPS (here, we call the resultant sequences ALLMAPS scaffolds), and 36 chromosome-scale scaffolds (more than 13 Mb) and 7 short-length scaffolds (chr37-chr43) were created, all with lengths less than 4 Mb (Supplementary Table 6). The contigs aligned on the 7 short-length scaffolds were excluded from further analysis and classified as unplaced scaffolds.

**Fig. 1.**
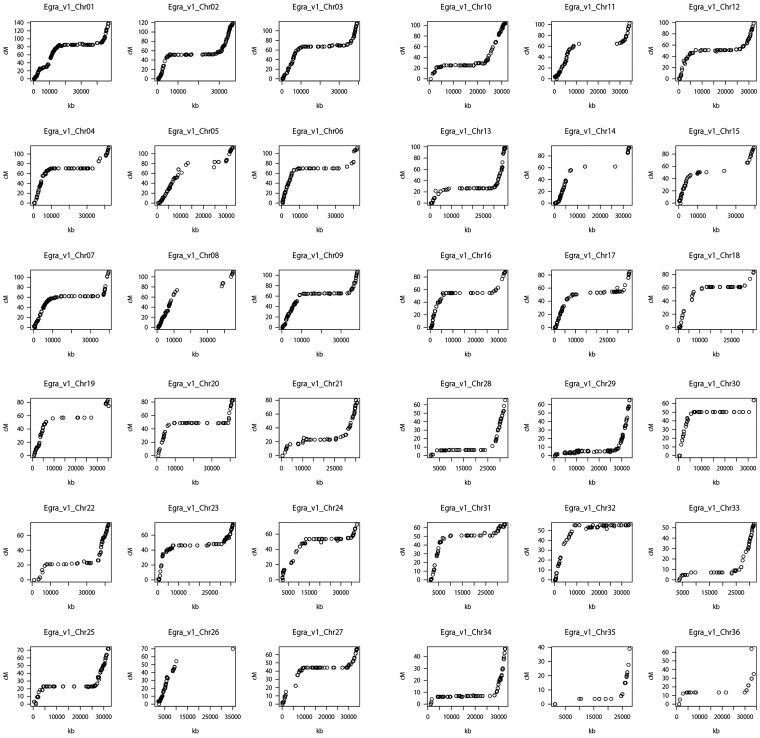
Correlation between physical and genetic distances on the *Eustoma* genome. The genetic distances were calculated from the 10B-581 F_2_ linkage map.

The 36 chromosome-scale Hi-C scaffold sequences were then aligned onto the ALLMAPS scaffolds by using Ragoo with the chimera cut option. After making several minor revisions manually, we created 36 pseudomolecules as sequences reflecting the 36 chromosomes in 10B-620 (Fig. 1, Supplementary Table 7 and Supplementary Table 8). In most of the chromosomes, the physical/linkage distances were clearly different between the end and central regions, and the differences suggested euchromatin and heterochromatin regions, respectively.

The 36 pseudomolecules and 64 unplaced scaffolds were designated “Egra_v1”. The total length of Egra_v1 is 1,324.8 Mb, with a gap length of 329,560 bp (Table 1, Supplementary Table 3). The total length of the 36 pseudomolecules is 1,308.6 Mb, which accounts for 98.8% of the assembled sequences. The corresponding chromosome numbers for the pseudomolecules are based on chromosome length from the longest to the shortest value. The lengths of the 36 pseudomolecules ranged from 47.12 Mb (Chr01) to 29.6 Mb (Chr36; Supplementary Table 9). When the total lengths were compared with the estimated genome size of 10B-620 (1587.6 Mb), all of the assembled scaffolds and the 36 pseudomolecules in Egra_v1 covered 83.4 and 82.4% of the genome, respectively. The Hi-C reads were mapped onto the 36 pseudomolecules by Juicer (https://github.com/aidenlab/juicer). A comparison with the contact map confirmed that the 36 scaffolds were appropriately created (Supplementary Figure 3).

**Table 1.** Statistics on the assembled Eustoma genome sequences and CDSs (egra_v1).

	Genome		Gene
	pseudomolecules +unplaced scaffolds	Pseudomolecules (Chr01-Chr36)	HC, CDS
Number of sequences	100	36	36,619
Total length (bp)	1,324,827,894	1,308,663,487	44,975,160
Average length (bp)	13,248,279	36,351,764	1,228
Maximum length (bp)	47,116,834	47,116,834	16,125
Minimum length (bp)	4,000	29,635,164	87
N50 length (bp)	35,699,481	35,699,481	1,680
Gaps (%)	0	0	0.006
GC%	37.9	37.9	44.0
BUSCOs (%) v3, obd10			
Complete	1,301 (94.6%)	1,277 (92.8%)	1,227 (89.2%)
Complete single-copy	1,110 (80.7%)	1,099 (79.9%)	1,046 (76.1%)
Complete duplicated	191 (13.9%)	178 (12.9%)	83 (13.1%)
Fragmented	21 (1.5%)	22 (1.6%)	83 (6.0%)
Missing	54 (3.9%)	76 (5.6%)	66 (4.8%)

The assembly quality of Egra_v1 was investigated by mapping the sequences onto 1,375 BUSCOs (Table 1, Supplementary Table 10a). The results demonstrated that the number of complete BUSCOs was 1,301 (94.6%), including 1,110 (80.7%) single-copy genes and 191 (13.8%) duplicated genes. There were 21 and 54 fragmented and missing BUSCOs, respectively. One BUSCO (ID = 39,084) was determined as a missing BUSCO on all Egra_v1 sequences; however, it was identified as a complete single BUSCO when BUSCO analysis was performed for the 36 pseudomolecule sequences (i.e. excluding unplaced scaffolds; Supplementary Table 10b). BLAST analysis was then performed for the missing 53 BUSCOs on Egra_v1 (Supplementary Table 10c), in order to investigate the presence or absence of significant similarity sequences on Egra_v1. A total of 43 BUSCOs showed partial sequence similarities against Egra_v1, while 10 BUSCOs did not hit for the Egra_v1 genome. We further investigated the existence of BUSCO sequences on the unmapped PE reads on Egra_v1 to examine the possibility of dropping the BUSCO sequences from the assembly. The Illumina PE reads (DRX302721) were mapped onto the Egra_v1 by Bowtie2. The mapping ratios were 99.4 and 99.1% in Read1 and Read2, respectively. Then, 942,398 (Read1) and 1,347,775 (Read2) unmapped reads were used for BLAST analysis against the 10 BUSCO sequences, for which similar sequences were not identified on Egra_v1 (Supplementary Table 10d). Two BUSCOs (6,551 and 26,742) were mapped with more than 228 reads, suggesting the existence of partial sequences on the Eustoma genome that were not assembled into Egra_v1. The other 8 BUSCOs were mapped with fewer than 54 reads, and thus these 8 BUSCO sequences were considered not to exist on the 10B-620 Eustoma genome.

We further investigated the assembly quality in Egra_v1 by using Merqury (Rhie *et al*. 2020; Supplementary Figure 4). The completeness and QV were estimated as 93.82% and 34.22, respectively. The Merqury results agreed with the Mercury results, and we concluded that ∼6% of the total genome sequences in 10B-620 were uncovered in Egra_v1. A large single peak and a gentle small peak were observed for the single and double copy sequences, respectively. Because the sequenced line, 10B-620, was an inbred line, it was considered that the double peak represented duplicated sequences that would be generated by ancient whole-genome duplication (WGD) events.

The total length of repetitive sequences in Egra_v1 was 975.1 Mb, which accounted for 73.6% of the assembled genome (Supplementary Table 11). Of the identified repetitive sequences, copia LTR (long terminal repeat) elements were the most frequently observed, occupying 30.1% of the assembled genome. The genomes of *C. canephora* (v1) (Denoeud *et al*. 2014) and *O. pumila* (Opu_r1.4) (Rai *et al.* 2021), which belong to the same order of *E. grandiflorum*, i.e. Gentianales, showed higher percentages of gypsy-type LTR elements than copia.

### Gene prediction and annotation

Iso-Seq sequences totaling 735.8 Mb and 982.2 Mb in length were obtained from leaves and young buds, respectively (Supplementary Table 1). The sequences from the two organs were integrated and clustered by Iso-Seq2, and 50,934 high-quality (hq) sequences were assembled (Supplementary Table 12). The 50,934 sequences were collapsed by mapping against the assembled sequence and filtered based on quality. The ORFs of the resultant 29,132 sequences were then predicted, and 11,175 nonduplicate full-length cDNA sequences were determined, with a total length of 14.0 Mb.

Meanwhile, de novo gene prediction was performed on the Egra_v1 genome sequences by using BRAKER2 with the *E. grandiflorum* transcript sequences listed in Supplementary Table 9. As a result, 202,561 candidate genes were predicted on the genome, with a total length of 242 Mb. The predicted gene sequences were merged with the 11,175 full-length cDNA sequences, and the resultant 200,998 sequences were regarded as the final set of predicted genes.

The 200,998 gene sequences were then classified as HC, LC, or TE based on evidence level. The numbers of predicted gene sequences classified as HC, LC, and TE were 36,619, 76,014, and 88,365, respectively (Supplementary Table 13, Table 1). The percentage of complete BUSCOs in HC was 89.2%, while those in LC and TE were 1.2 and 1.5%, respectively. Therefore, most of the protein-coding gene sequences were designated as HC.

Functional gene annotation was performed by using a modified version of Hayai annotation with refereeing through the Kusaki database (http://pgdbjsnp.kazusa.or.jp/app/kusakidb). The numbers of the functional annotated genes in HC, LC, and TE were 25,936, 16,929, and 54,565, respectively. The annotation results of the 25,936 HC genes are listed in Supplementary Table 14. The species listed most frequently as top-hit species against the *E. grandiflorum* genes (HC) were *Coffea arabica* (40.3%), followed by *C. canephora* (9.1%) and *C. eugenioides* (3.4%). The most frequently observed top-hit families were Rubiaceae, Solanaceae, and Nyssaceae, which made up 53.1%, 8.0%, and 5.5% of the top-hit family list, respectively (Supplementary Table 15). There were 10,356 HC genes annotated with GO and GOSLIM-PIR terms, 13,205 with PFAM, 13,826 with InterPro, and 1,467 with EC (Supplementary Table 16, Supplementary Table 17, Supplementary Table 18, Supplementary Table 19, and Supplementary Table 20).

### Comparison with other plant species at the genome sequence level

The genome structure of *E. grandiflorum* was compared with those of *G. dahurica* (Gda; Li *et al*. 2022), *C. canephora* (v1; Denoeud *et al.* 2014), and *O. pumila* (Opu_r1.4; Rai *et al.* 2021) by aligning homologous sequence pairs along each pseudomolecule. Partial syntenic relationships were observed between *E. grandiflorum* and *G. dahurica*, which belongs to the family Gentianaceae, but there were no clear similarities at the chromosome level (Supplementary Figure 5).

Interestingly, more partial syntenic relationships were observed between the genomes of *E. grandiflorum* and *C. canephora* or *O. pumila* than between the genomes of *E. grandiflorum* and *G. dahurica* (Supplementary Figure 6). It was particularly noteworthy that duplicated syntenies were observed on *E. grandiflorum* against several specific regions on the genomes of *C. canephora* or *O. pumila*. For example, the genome sequence in the first half of chr6 in *C. canephora* was similar to those of chr5, 14, 18, 19, 20 21, and 32 in *E. grandiflorum*.

Li *et al.* (2022) predicted that a WGD event would occur on the genome of *G. dahurica* after whole-genome triplication (WGT) of eudicots (WGT-γ) (Jaillon *et al.* 2007) and showed 1:4 gene duplication between *C. canephora and G. dahurica*. Our results agreed with those of Li *et al*. (2022) and showed additional duplication that suggested a possibility of further WGD after the divergence between *E. grandiflorum* and *G. dahurica*.

### Comparison with other plant species at the gene level

**Fig. 2.**
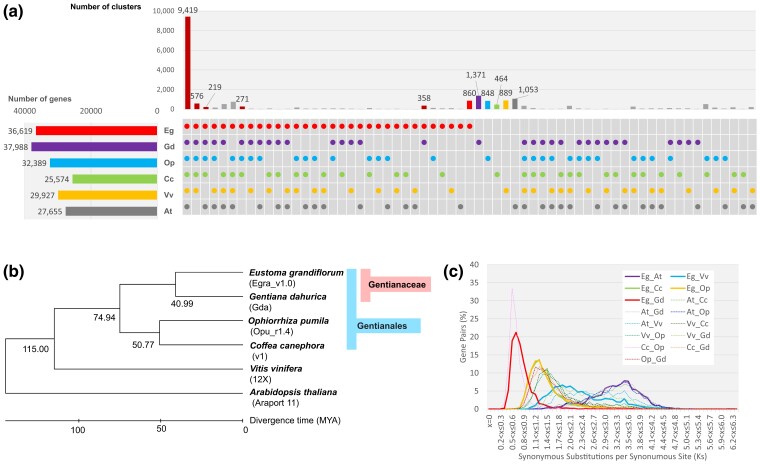
Comparative and phylogenetic analyses with other plant species at the gene level. a) Number of gene clusters created with genes of *E. grandiflorum* (Ev), G. *dahurica* (Gd), *O. pumila* (Op), c*) canephora* (Cc), *V. vinifera* (Vv), and *A. thaliana* (At). b) A phylogenetic tree of 1,729 common single-copy genes of the 6 plant species. c) Distribution of Ks values of orthologous gene pairs in *E. grandiflorum* and the other 5 species.

The translated protein sequences in the HC gene sequences were clustered and compared with the protein sequences in other plant species (*G. dahurica*, *O. pumila*, *C. canephora*, *V. vinifera*, and *A. thaliana*) at the amino acid level by OrthoFinder v2.5 (Emms and Kelly 2019). The 36,619 HC genes in Egra_v1 were classified into 18,386 clusters, and 836 of these clusters (consisting of 9,340 genes or 25.5% of the total), were specific to *E. grandiflorum* (Fig. 2a, Supplementary Table 21). The number of species-specific clusters in *E. grandiflorum* was similar to those in *O. pumila* and *V. vinifera* and less than that in *G. dahurica*. A total of 18,386 (50.2%) of the *E. grandiflorum* genes were clustered with all 5 plant species compared.

Three of the five compared species, i.e. *G. dahurica*, *O. pumila*, and *C. canephora*, belong to the order Gentianales, to which *E. grandiflorum* also belongs, while *V. vinifera* and *A. thaliana* belong to the orders Vitales and Brassicales, respectively. A total of 576 clusters were created with the genes commonly observed in Gentianales and *V. vinifera*, which was 2.6-fold the number of clusters in the common genes in Gentianales and *A. thaliana*. These results suggested that *V. vinifera* was closer to the species in the order Gentianales than to those in *A. thaliana*. A total of 271 clusters were created with the genes commonly observed in the four species belonging to Gentianales. The number of clusters created with the common genes between the two species in the family Gentianaceae was 358.

Phylogenetic analysis was further performed with 1,729 single-copy genes observed in *E. grandiflorum*, *G. dahurica*, *O. pumila*, *C. canephora*, *V. vinifera*, and *A. thaliana*; *A. thaliana* was used for the outgroup (Fig. 2b). Of the four species in the order Gentianales, *C. canephora* and *O. pumila* belong to the family Rubiacea. Our results suggested that the families Gentianaceae and Rubiaceae diverged ∼74.94 MYA. Li *et al.* (2022) reported that the two families diverged 64.22 MYA, 10.72 MYA later than in our result. We estimated the year of divergence based on a neighbor-joining phylogenetic tree, while Li *et al*. (2022) used an MCMCTree. In addition, different sets of single-copy genes were used in the two studies. The difference in methods and gene sets wdifference in diverged year estimation. In the family Gentianaceae, divergence between the genera *Eustoma* and *Gentiana* was estimated to have occurred ∼40.99 MYA. Ks values were also calculated for the estimation with 1,729 single-copy genes. The distributions of Ks values were supported by the phylogenetic relations of the six species (Fig. 2c).

### Diversity analysis in 9 commercial varieties

Illumina PE reads of the 9 *E. grandiflorum* varieties bred by Japanese commercial companies were mapped onto Egra_v1 to detect base variants. A total of 16,412,137 candidate variants were identified and filtered according to the following conditions: QUAL 250 ≤, DP 10 ≤, GQ 10 ≤, max-missing = 0.8, and excluding MAF (minor allele frequency) = 0 or 0.5. The remaining number of variants was 254,205. The base variant density on Egra_v1 is shown in Supplementary Figure 7. In most of the chromosomes, fewer variants were observed in the approximately range of 30–70% of the median. However, a few chromosomes, such as Chr5 and Chr31, showed more variants in the approximately range of 30–70% of the median. Phylogenetic analysis showed that “Borelo white” was genetically distant from the other 8 varieties (Supplementary Figure 8).

## Conclusion

In this study, we established a chromosome-scale genome assembly of *E. grandiflorum*, the first complete genome sequence in the genus *Eustoma*. The assembled genome covered 83.4% of the estimated genome, and the 36 pseudomolecules occupied 98.8% of the assembled genome. In addition, a total of 36,619 protein-coding genes were identified on the assembled genome with high confidence. The resultant genome assembly will be useful for genetic and genomic studies and will deepen our understanding of the species in the genus *Eustoma* and the family Gentianaceae.

## Supplementary Material

jkac329_Supplementary_Data

## Data Availability

The assembled genome sequences have been submitted to the DDBJ/ENA/NCBI public sequence databases under the BioProject ID PRJDB12119. The assembled genome and gene sequences, the SNPs of the 9 commercial varieties, and the MST map information are available at Plant GARDEN (https://plantgarden.jp/en/list/t52518). Supplementary tables and figures are available at figshare: https://doi.org/10.25387/g3.20382279. Supplemental material is available at G3 online.

## Notes

*Communicating editor: J. Udall*
