# Supplementary material for: Chromosome-scale genome assembly of Eustoma grandiflorum, the first complete genome sequence in the genus Eustoma
Source: G3 (Bethesda). 2022 Dec 19;13(2):jkac329. doi: 10.1093/g3journal/jkac329 (PMC9911058; doi:10.1093/g3journal/jkac329)
Supplement: jkac329_Supplementary_Data [file jkac329_supplementary_data.zip › Supplementary Figures.pdf]

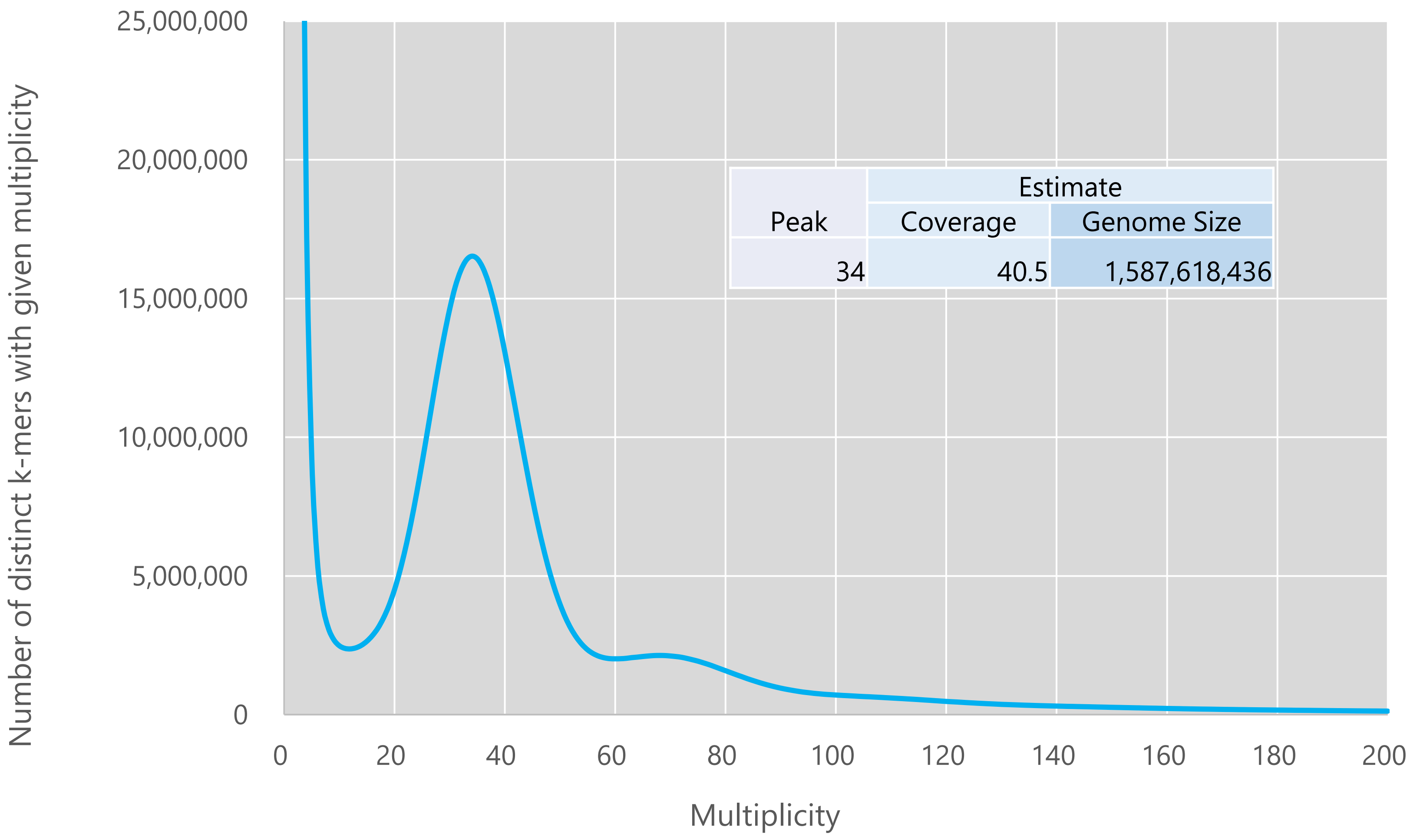

Fig. S1. Genome size estimation using Jellyfish with the distribution of the number of distinct kmers (kmer = 17) with the given multiplicity values.

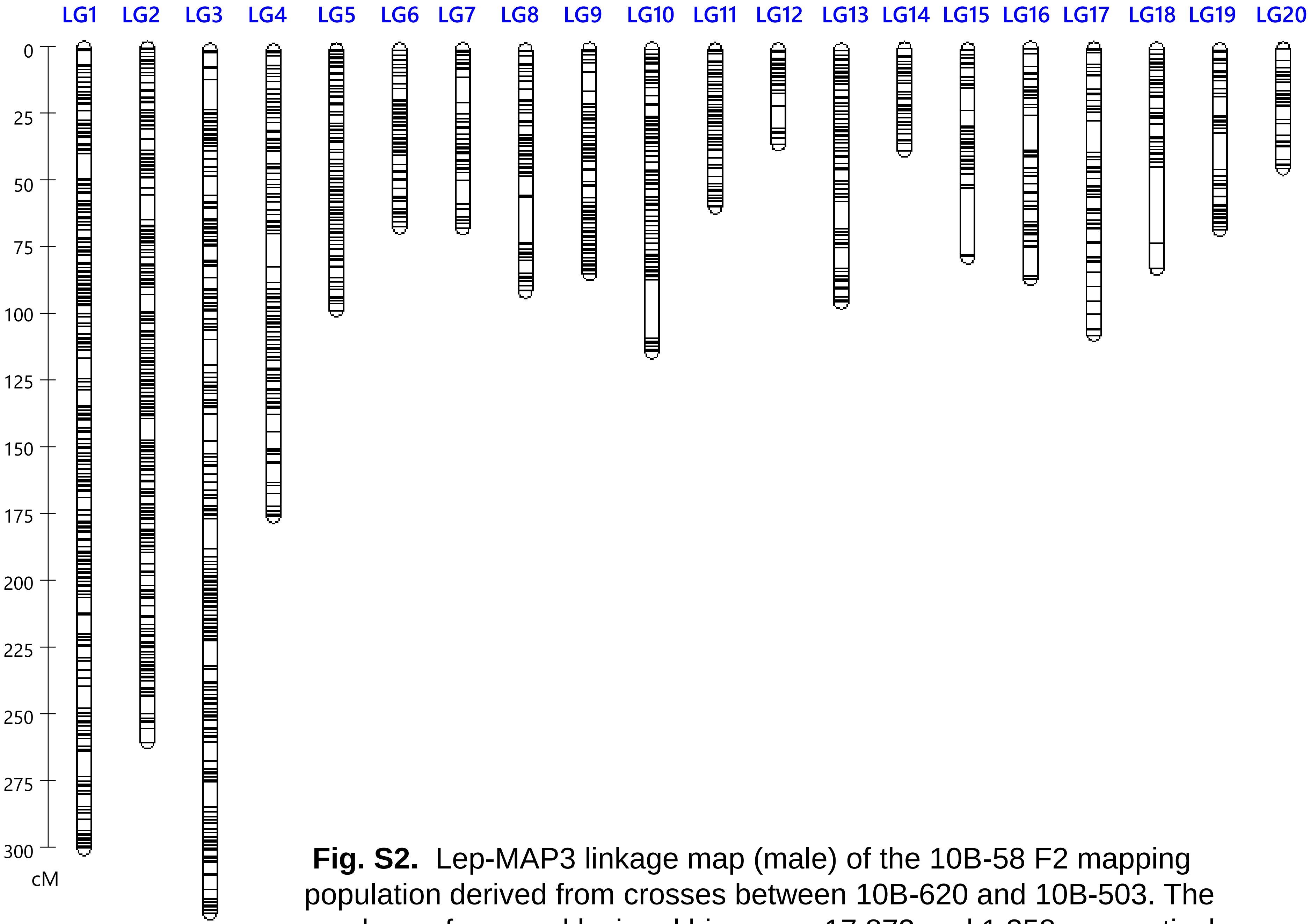

Fig. S2. Lep-MAP3 linkage map (male) of the 10B-58 F2 mapping population derived from crosses between 10B-620 and 10B-503. The numbers of mapped loci and bins were 17,873 and 1,358, respectively.

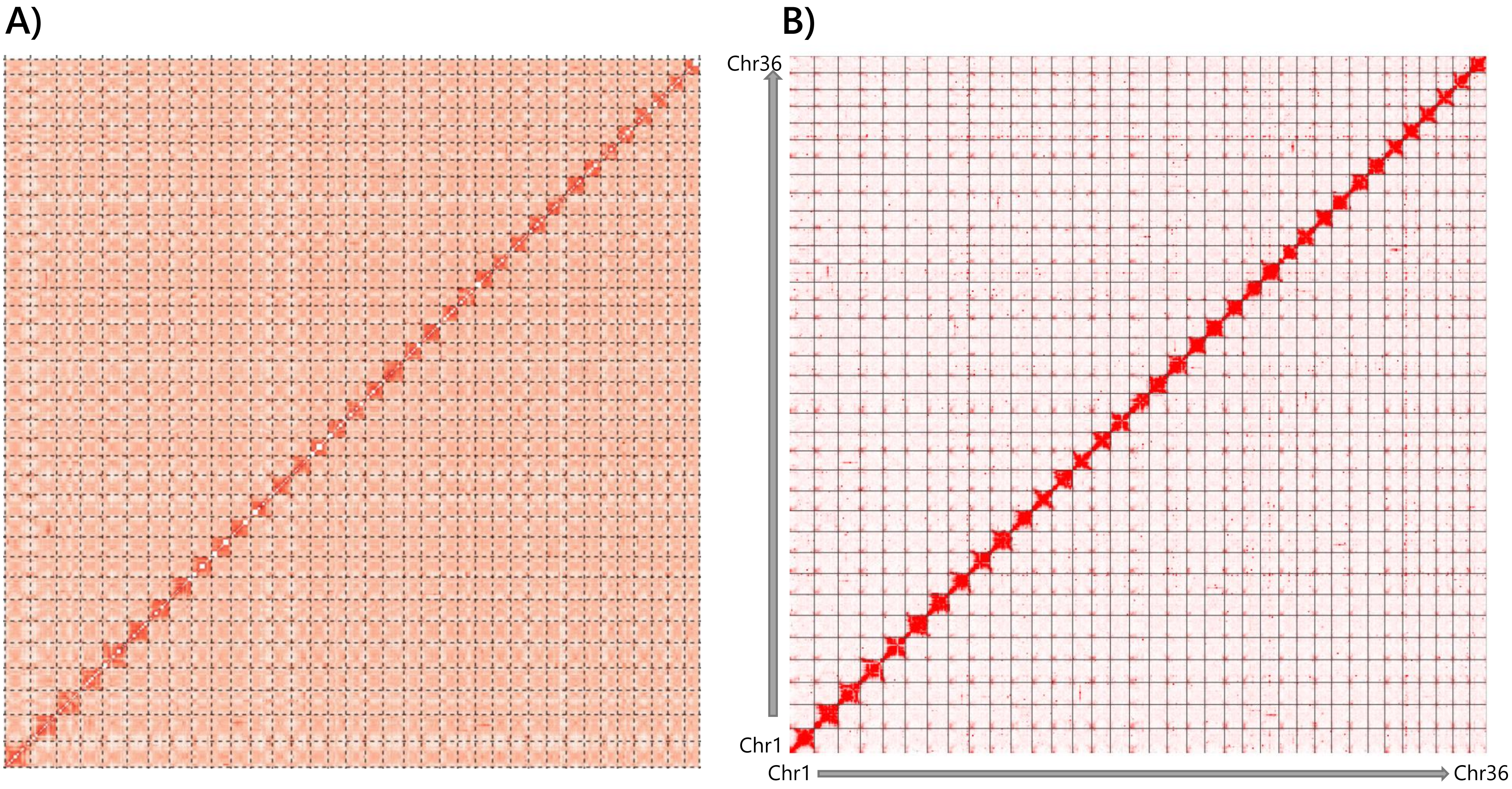

Fig. S3. Hi-C contact maps for the 36 pseudomolecules created by Juicebox. A) 36 pseudomolecules (chromosome-level scaffolds) created by Proximo Hi-C. Juicebox was used for creating the map. B) 36 pseudomolecules of Egra_v1.0. Hi-C was mapped onto the pseudomolecules by juicer.

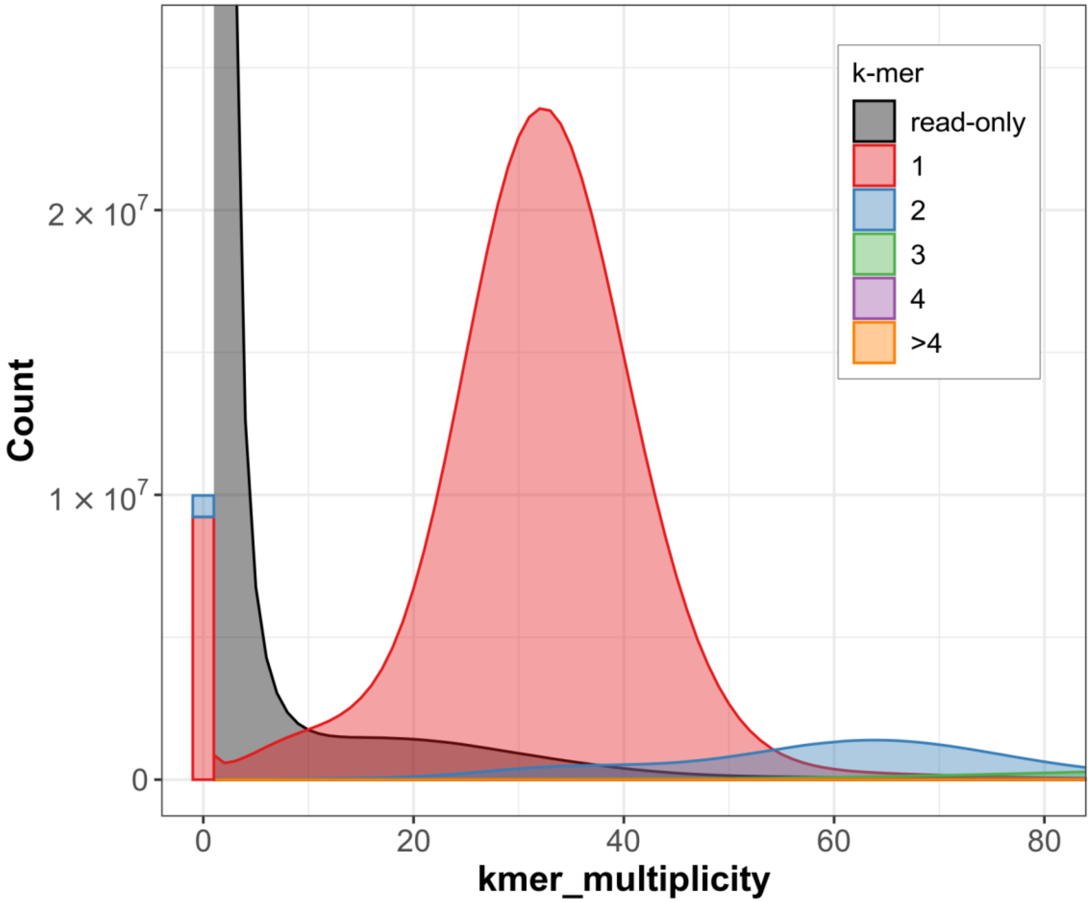

Sequence	*k*-mers uniquely found only in the assembly	*k*-mers found in both assembly and the read set	QV	Error rate	k-mer set used for measuring completeness	solid k-mers in the assembly	Total solid k-mers in the read set	Completeness (%)
Egra_v1	9,978,606	1,323,992,553	34.22	0.00037819	all	587,888,846	626641758.00	93.82

Fig. S4. Merqury copy number spectrum plots for the assembled genome, Egra_v1 (including 36 chr and unplaced scaffolds). The red, blue, green, purple and orange plots represent kmer multiplicities peaks with x1, x2, x3, x4 and x >4 copy sequences in Egra_v1, respectively. The gray plot represents kmers identified only on paired end reads of the sequenced line, 10B-620.

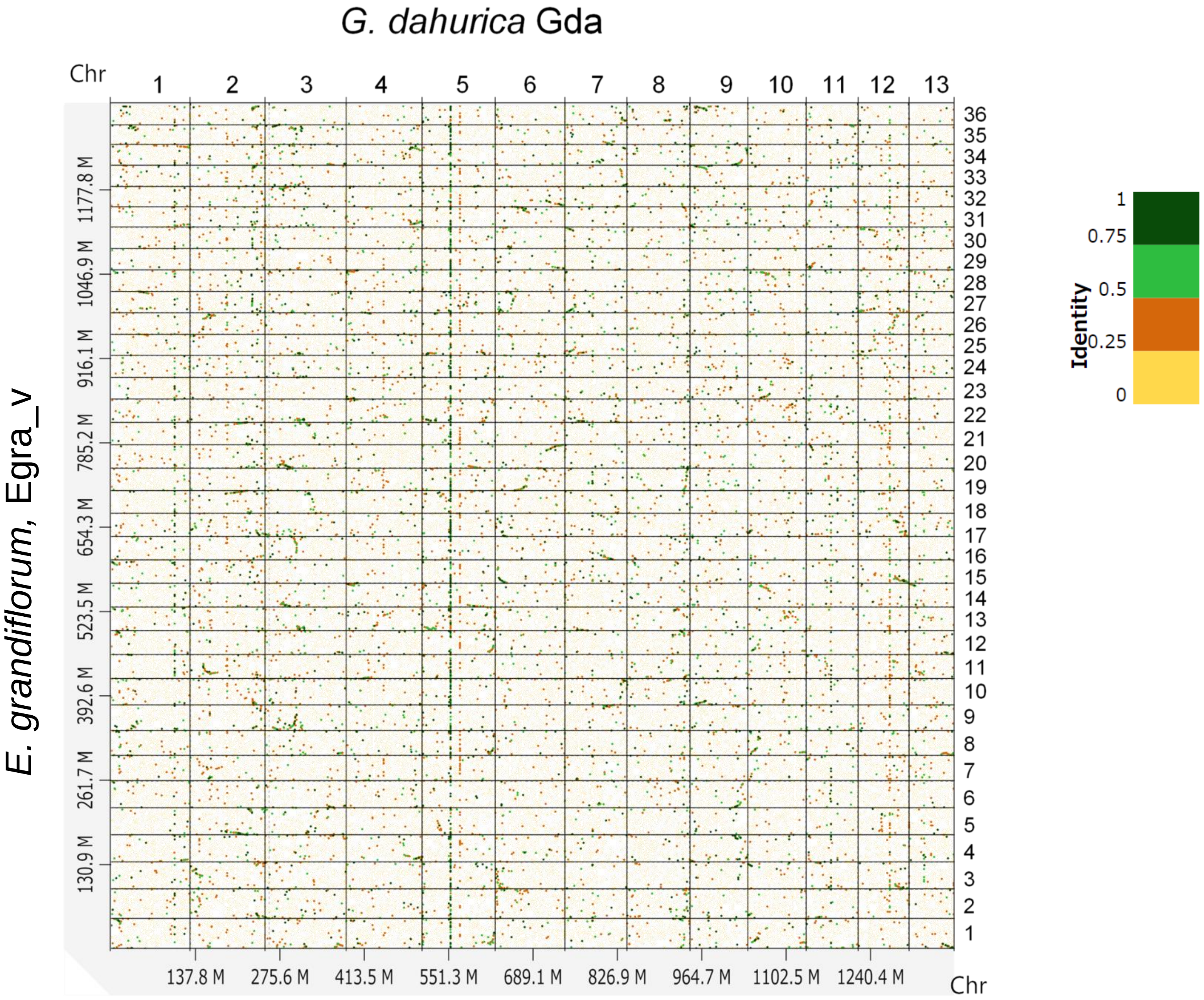

Fig. S5. Graphical view of syntenic relationships between *E. grandiflorum* and *G. dahurica.*

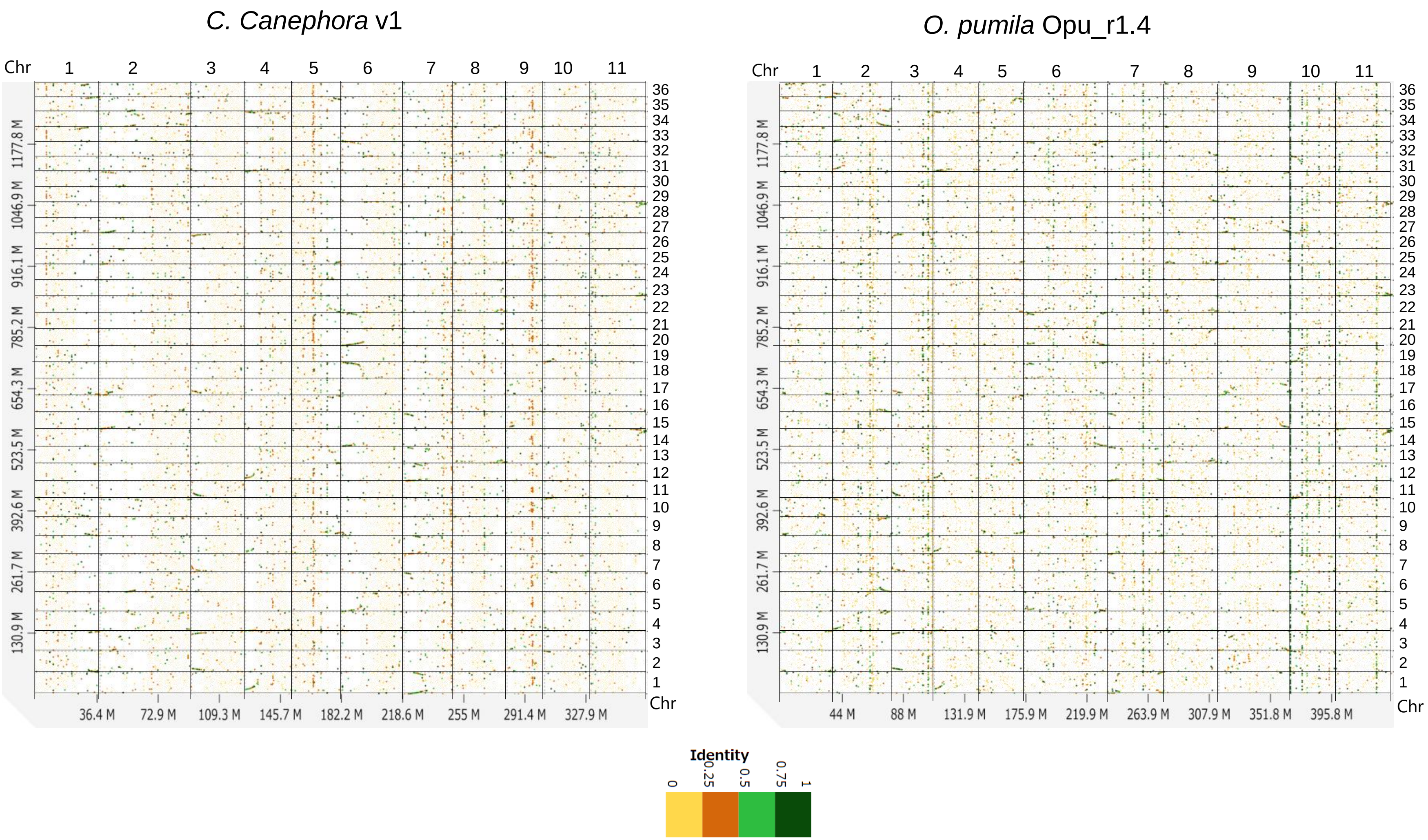

Fig. S6. Graphical view of syntenic relationships between *E. grandiflorum* and *C. canephora* (left) or *O. pumila* (right).

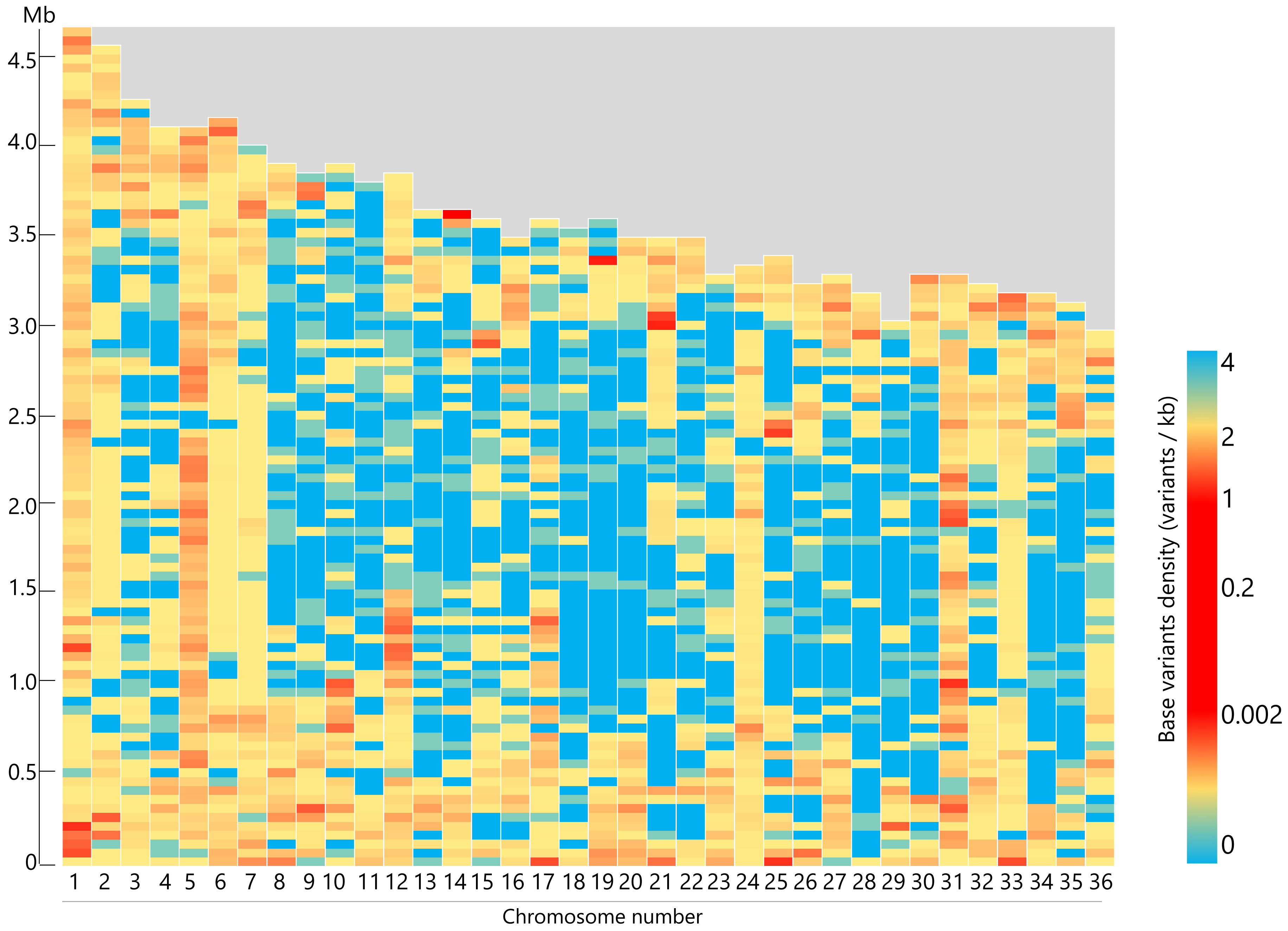

Fig. S7. Base variants density on the Eustoma genome. The variants were identified with nine Eustoma varieties, and the density (variants /kb) was calculated in each 500kb windows.

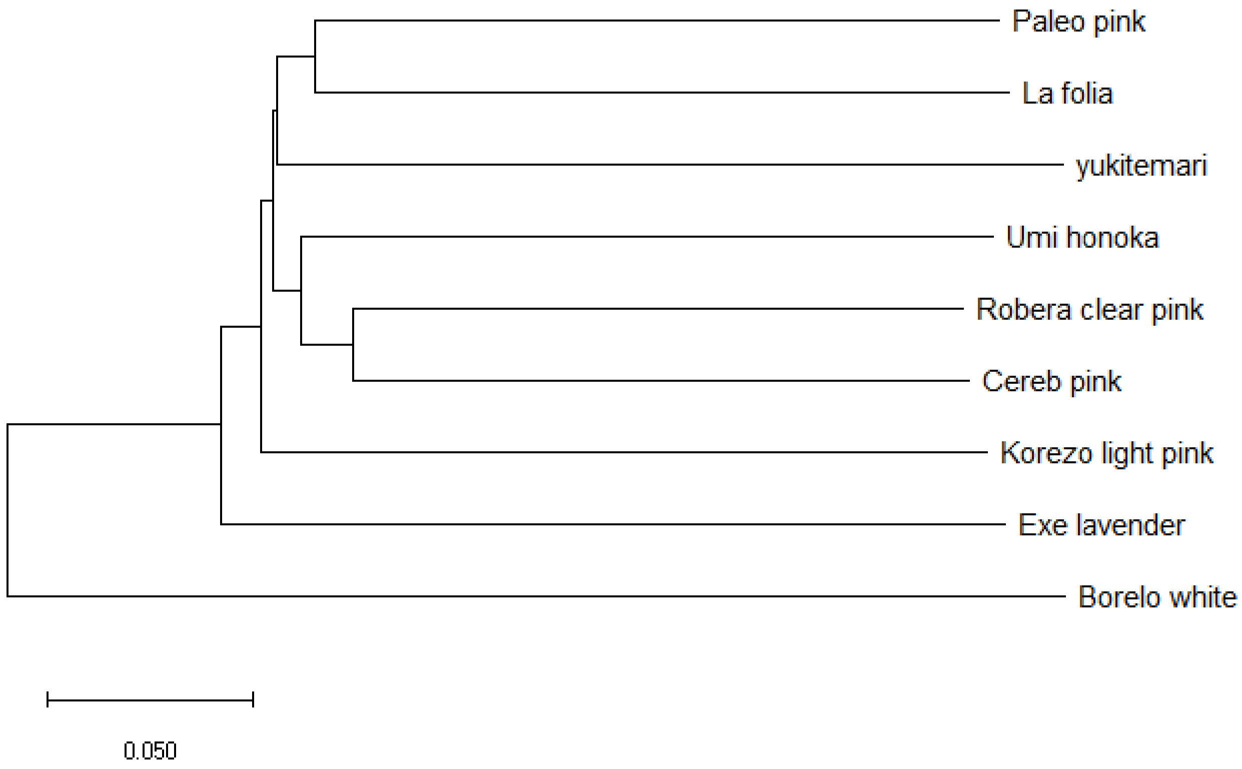

Fig. S8. A phylogenetic tree of nine Eustoma varieties based on 254,205 variants.
